# Anatomical Rationale for Motor Endplate Zone-Targeted Botulinum Toxin Injection in the Masseter Muscle: A Hypothesis-Generating Review

**DOI:** 10.3390/toxins18070304

**Published:** 2026-07-14

**Authors:** Kazuya Yoshida

**Affiliations:** Department of Oral and Maxillofacial Surgery, National Hospital Organization, Kyoto Medical Center, 1-1 Mukaihata-cho, Fukakusa, Fushimi-ku, Kyoto 612-8555, Japan; yoshida.kazuya.ut@mail.hosp.go.jp; Tel.: +81-75-641-9161; Fax: +81-75-643-4325

**Keywords:** botulinum toxin, masseter muscle, motor endplate zone, bruxism, temporomandibular disorders, injection technique, adverse effects

## Abstract

Botulinum neurotoxin (BoNT) injection into the masseter muscle is used in selected conditions associated with excessive or painful masseter activity, including oromandibular dystonia, masseter-related myalgia, and bruxism-associated masticatory muscle pain. However, clinical outcomes and adverse-event profiles vary widely, partly because injection technique, dose, dilution, injection depth, anatomical targeting, patient selection, and injector experience have not been consistently standardized. This review examines the anatomical and pharmacological rationale for motor endplate zone (MEZ)-targeted BoNT injection into the masseter muscle. MEZ-targeted injection is not presented as an established clinical guideline or as a proven superior technique but as a hypothesis-generating framework for future controlled studies. Anatomical and electrophysiological studies indicate that neuromuscular junctions in the masseter muscle are concentrated within relatively restricted MEZs. From a pharmacological perspective, delivery of BoNT close to these zones may allow more efficient neuromuscular blockade with lower doses, although direct clinical evidence demonstrating superiority over conventional techniques remains insufficient. Reported adverse events may be influenced by excessive dosing, inaccurate injection depth, diffusion into adjacent structures, or repeated high-dose injections. Future studies should evaluate anatomically informed, dose-minimized injection strategies using clearly defined clinical populations, standardized diagnostic criteria, detailed reporting of injection technique, objective functional outcomes, and systematic adverse-event assessment.

## 1. Introduction

Botulinum neurotoxin (BoNT) has been used in selected conditions involving excessive or painful activity of the masseter muscle, including oromandibular dystonia, masseter-related myalgia, and bruxism-associated masticatory muscle pain [1,2,3,4]. Traditionally, the therapeutic effects of BoNT have been attributed to the inhibition of acetylcholine release at the neuromuscular junction, resulting in reduced muscle activity and alleviation of excessive muscle contraction [5,6]. In addition to its well-established effects on motor nerve terminals, accumulating evidence indicates that BoNT may exert analgesic effects through modulation of sensory afferent pathways [7,8]. Experimental and clinical studies have suggested that BoNT can suppress the release of pain-related neuropeptides, such as substance P and calcitonin gene-related peptide, from peripheral sensory nerve endings, thereby reducing peripheral sensitization and nociceptive transmission [7,8,9].

Temporomandibular disorders (TMDs) do not represent a single diagnosis but rather an umbrella term encompassing heterogeneous musculoskeletal and joint-related conditions. Similarly, bruxism is currently regarded as a masticatory muscle activity or behavior rather than a disease entity [10,11,12,13]. Therefore, BoNT should not be considered a generic treatment for either TMDs or bruxism. In the present review, these conditions are discussed only when excessive or painful masseter activity is clinically relevant.

Oromandibular dystonia differs from bruxism-associated muscle activity and myogenous pain conditions in that it is a neurological movement disorder for which BoNT is more firmly established as a therapeutic option [3,14,15,16]. Nevertheless, even in oromandibular dystonia, accurate muscle selection, dose minimization, appropriate injection depth, and avoidance of diffusion into adjacent structures remain essential for optimizing benefits and reducing adverse effects [3,14,15,16].

Since the early 2000s, numerous clinical studies have evaluated BoNT for myofascial pain related to TMDs and bruxism-associated masticatory muscle pain [2,4,17,18,19,20,21,22,23,24,25,26,27,28,29,30,31,32]. However, systematic reviews, meta-analyses, and umbrella reviews have reported inconsistent conclusions regarding efficacy and safety [33,34,35,36,37,38,39,40,41,42,43,44,45,46,47,48,49,50,51]. Some studies have reported greater pain reduction compared with control groups [4,20,24,25,29], whereas others have found no significant superiority over placebo or conservative therapies [17,19,21,22,23]. Concerns have also been raised regarding adverse effects, including masticatory weakness, facial asymmetry, and potential mandibular bone changes [46,50,51,52,53].

One possible explanation for these inconsistent findings is that BoNT injection into the masseter muscle has often been evaluated as a uniform intervention, despite substantial heterogeneity in patient selection, diagnosis, dose, dilution, injection depth, target muscle layer, anatomical targeting, and injector experience [52,54,55,56]. In neurological applications of BoNT, detailed anatomical knowledge and precise localization of target zones, including motor endplate zones (MEZs), are considered important for optimizing therapeutic effects and minimizing unnecessary diffusion [54,55,56]. In contrast, studies involving the masticatory muscles have not always reported injection technique and anatomical targeting in sufficient detail [52,54,55,56].

Anatomical and electrophysiological studies indicate that neuromuscular junctions in the masseter muscle are not uniformly distributed but are concentrated within relatively restricted MEZs [57,58,59,60,61,62,63,64]. Because BoNT acts at the neuromuscular junction, delivery of the toxin near these zones is pharmacologically rational and may allow more efficient neuromuscular blockade with lower doses [5]. However, direct clinical evidence demonstrating the superiority of MEZ-targeted masseter injection over conventional injection techniques remains insufficient.

The aim of this review is to examine the anatomical and pharmacological rationale for MEZ-targeted BoNT injection into the masseter muscle and to propose a hypothesis-generating framework for future controlled studies in selected conditions involving excessive or painful masseter activity.

## 2. Clinical Context: Conditions Involving Excessive or Painful Masseter Activity

BoNT injection into the masseter muscle is a muscle-directed intervention rather than a disease-specific treatment. Its clinical relevance depends on the underlying condition, the contribution of the masseter muscle to symptom generation, and the therapeutic goal. Therefore, BoNT should not be discussed as a generic treatment for TMDs or bruxism but as a potential intervention for selected patients with clinically relevant excessive or painful masseter activity [3,10,11,12,13,14,15].

### 2.1. Oromandibular Dystonia

Oromandibular dystonia is a focal movement disorder characterized by involuntary, sustained, or repetitive contractions of the masticatory, lingual, perioral, or lower facial muscles [3,14,15]. When the jaw-closing muscles are predominantly involved, excessive activity of the masseter and temporalis muscles may cause jaw clenching, trismus, tooth wear, muscle pain, impaired mastication, speech disturbance, and difficulty with oral hygiene or dental treatment. In such cases, the masseter muscle is often one of the principal therapeutic targets [3,14,15].

In contrast to bruxism or heterogeneous myogenous pain conditions, oromandibular dystonia is a neurological disorder for which BoNT is widely regarded as an established therapeutic option [3,14,15,16]. The therapeutic goal is not simply analgesia but reduction in involuntary muscle contraction and restoration of oral function [3,15,16]. Nevertheless, treatment response depends on accurate identification of the involved muscles, appropriate dose selection, injection depth, and avoidance of diffusion into adjacent structures. Excessive weakening of the jaw-closing muscles may impair mastication, whereas inaccurate injection may fail to suppress dystonic activity or may induce adverse effects such as facial asymmetry, dysphagia, or speech changes [3,15,16].

Oromandibular dystonia provides an important model for considering anatomical precision in masseter injection. Even when BoNT is clearly indicated, efficacy and safety depend on accurate delivery to the functionally relevant region of the target muscle. Thus, MEZ-targeted injection may be relevant as a conceptual framework for improving injection accuracy and dose efficiency in jaw-closing dystonia, although superiority over conventional techniques remains to be tested [54,57,58,59,60,61,62,63,64].

### 2.2. Masseter-Related Myalgia and Myogenous TMD

Temporomandibular disorders are not a single diagnosis but an umbrella term encompassing heterogeneous musculoskeletal and joint-related conditions affecting the temporomandibular joints, masticatory muscles, and associated structures [10,65]. Therefore, the expression “BoNT treatment for TMDs” is imprecise unless the specific diagnostic subgroup and symptom generator are clearly defined [10,65]. In the context of masseter injection, the most relevant subgroup is masseter-related myalgia or myogenous TMD, in which pain is localized to the masseter muscle and is associated with tenderness, functional limitation, or increased muscle activity [4,10,16,52].

The potential rationale for BoNT in such patients is based on both motor and sensory mechanisms [5,7,8,9]. By reducing excessive muscle activity, BoNT may decrease sustained contraction and mechanical loading of painful muscle tissue. In addition, BoNT may modulate peripheral nociceptive mechanisms by inhibiting the release of pain-related neuropeptides from sensory nerve endings [7,8,9]. However, masseter-related myalgia is multifactorial, and pain may be influenced by psychosocial factors, central sensitization, parafunctional activity, sleep disturbance, occlusal factors, joint pathology, or coexisting orofacial pain conditions [10,57]. Therefore, BoNT should not be regarded as a routine or first-line treatment for myogenous TMD [3,10,14].

In clinical practice and future studies, careful patient selection is essential [3,10,52]. BoNT may be considered only in selected patients with persistent masseter-related myalgia after appropriate conservative management, especially when excessive masseter activity is considered to contribute substantially to symptoms. Diagnostic clarity is important because patients with arthrogenous TMD, neuropathic pain, headache disorders, inflammatory disease, or centrally mediated pain may not benefit from masseter-directed chemodenervation [3,14]. For this reason, future trials should clearly define diagnostic criteria, pain localization, baseline muscle activity, bite force, previous conservative treatments, and functional outcomes [3,10,14].

### 2.3. Bruxism-Associated Masticatory Muscle Pain

Bruxism is currently defined as masticatory muscle activity or behavior rather than a disease entity [11,12,13]. Sleep bruxism and awake bruxism may occur in healthy individuals and do not necessarily require treatment [11,12,13]. Therefore, BoNT should not be considered a generic treatment for bruxism itself. The indication for intervention should be based on clinically relevant consequences, such as persistent masticatory muscle pain, functional impairment, severe tooth wear, prosthodontic complications, or other tissue damage, rather than on the mere presence of bruxism activity [11,12,13].

This distinction is particularly important when discussing BoNT injection into the masseter muscle. BoNT may reduce the intensity of masseter contraction, but it does not necessarily eliminate the underlying behavior, arousal-related events, psychosocial contributors, or central mechanisms associated with bruxism [11,12,13]. Consequently, apparent improvements in pain or muscle fatigue may not be equivalent to “treatment of bruxism” itself. The therapeutic target should instead be described more precisely as bruxism-associated masticatory muscle pain or clinically significant excessive masseter activity [11,12,13].

Future studies should incorporate standardized bruxism assessment frameworks and distinguish self-reported, clinically assessed, and instrumentally confirmed bruxism [66]. Outcomes should include pain, jaw function, bite force, electromyographic activity, oral health consequences, sleep-related variables when relevant, patient-reported outcomes, and adverse events. These distinctions are essential to avoid overestimating the role of BoNT in bruxism and to identify patients in whom masseter injection may be clinically meaningful [3,14,66].

### 2.4. Ethical Considerations and Avoidance of Overtreatment

Because TMDs and bruxism are heterogeneous constructs, inappropriate labeling may lead to overdiagnosis, overtreatment, and unnecessary medicalization [65,67]. This issue is particularly important for BoNT therapy, which is invasive, costly, and associated with dose- and diffusion-related adverse effects [46,50,51,68,69,70,71,72,73,74,75,76,77,78,79,80,81]. Therefore, BoNT should not be promoted as a routine, cosmetic, preventive, or first-line intervention for TMDs, bruxism, or nonspecific jaw discomfort [10,65,67].

Conservative, reversible, and patient-centered management should remain the initial approach for most patients with TMD-related pain or bruxism-associated symptoms [3,10,14,65]. Such management may include patient education, behavioral modification, physical therapy, oral appliances when appropriate, pharmacological pain management, sleep-related assessment, and treatment of contributing psychosocial or systemic factors. BoNT should be considered only after careful diagnosis, adequate conservative care, and individualized risk–benefit assessment [3,10,14,65].

Potential adverse effects of masseter BoNT injection should be explained clearly to patients, as discussed in detail in Section 5 [46,50,51,68,69,70,71,72,73,74,75,76,77,78,79,80,81]. Ethical use requires dose minimization, avoidance of unnecessary repeated injections, functional monitoring, and transparent discussion of the limitations of current evidence [15,46,50,51,68,69,70,71,72,73,74,75,76,77,78,79,80,81].

Thus, the proposed MEZ-targeted framework should not be interpreted as an encouragement to expand indications for BoNT [10,65,67]. Rather, it is intended to improve anatomical precision and reduce unnecessary toxin exposure when BoNT is clinically justified. Future controlled studies should evaluate whether anatomically informed, dose-minimized injection strategies can improve the balance between benefit and risk in clearly defined patient populations.

## 3. Anatomical Basis for Masseter Injection

The masseter muscle is a thick, quadrilateral masticatory muscle that plays a central role in mandibular elevation, forceful biting, and stabilization of the mandible during oral function [82,83,84,85,86]. Because of its superficial location and relative ease of palpation, the masseter is often considered an accessible target for BoNT injection. However, its layered structure, variable thickness, regional functional differences, and close anatomical relationship to adjacent structures require careful consideration when planning injection sites, depth, and dose distribution [82,83,84,85,86,87,88,89].

### 3.1. Layered Structure of the Masseter Muscle

The masseter muscle has traditionally been described as consisting of superficial and deep layers [82,83]. The superficial layer arises mainly from the zygomatic arch and zygomatic process and descends posteroinferiorly to attach to the angle and lateral surface of the mandibular ramus [82,83,84,85,86]. The deep layer is located more posteriorly and superiorly, with fibers running more vertically from the zygomatic arch to the upper portion of the mandibular ramus and coronoid region. Some anatomical descriptions further subdivide the muscle into superficial, intermediate, and deep layers, reflecting the complex internal architecture of the masseter [84,85,86].

This layered organization is clinically relevant because the superficial and deep portions may differ in fiber direction, thickness, functional contribution, and susceptibility to toxin diffusion [85,86]. Superficial injections may primarily affect the more accessible outer portion of the muscle, whereas deeper injections may influence the deep layer or may increase the risk of spread toward adjacent deep structures if performed inaccurately [87,88,89]. Therefore, injection depth should not be determined by a fixed value alone but should be individualized according to muscle thickness, subcutaneous tissue, patient sex, body habitus, degree of hypertrophy or hyperactivity, and the intended target layer [85,86,87,88,89].

In patients with jaw-closing dystonia, masseter hypertrophy, or marked clenching activity, both superficial and deeper regions of the muscle may contribute to excessive contraction [3,14,15,90]. In contrast, in some patients with masseter-related myalgia or bruxism-associated masticatory muscle pain, modulation of the superficial or central portion of the muscle may be sufficient [10,11,12,13,52]. These differences underscore the importance of linking anatomical targeting to the clinical objective of treatment [3,14,15,90].

### 3.2. Surrounding Anatomical Structures Relevant to Safety

**Figure 1:**
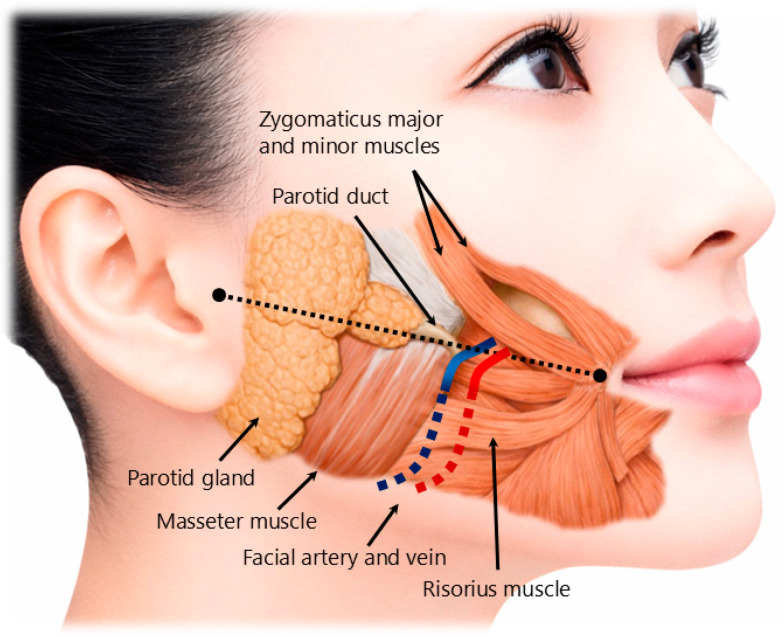
Anatomical structures relevant to safe BoNT injection into the masseter muscle. The red and blue lines indicate the facial artery and facial vein, respectively. The black dotted line connects the oral commissure and the tragus and indicates the upper boundary of the generally recommended injection region; injections are typically placed below this line to reduce the risk of diffusion into adjacent facial muscles.

The masseter muscle is adjacent to several structures that may be affected by inaccurate injection or unintended diffusion of BoNT [87,88,89] (Figure 1). The parotid gland lies posterior and superficial to the posterior border of the masseter, and its anterior extension may vary among individuals [87,88,89]. Excessively posterior or posterior–superior injection may increase the risk of diffusion toward the parotid gland, potentially contributing to xerostomia [68,70,89].

The facial artery and vein pass near the anterior border of the masseter muscle [86,87,88,89] (Figure 1). Therefore, injections placed too far anteriorly or repeated needle passes in this region may increase the risk of vascular injury, bruising, hematoma, or perioral swelling [68,70,71]. In addition, several muscles of facial expression, including the risorius, zygomaticus major and minor, depressor anguli oris, and lateral portion of the orbicularis oris, are located anterior or superficial to the masseter region [86,87,88,89] (Figure 1). Diffusion into these muscles may result in smile asymmetry or other unwanted facial changes [68,70,73,74].

Medially and deeply, the masseter is anatomically related to the mandibular ramus, medial pterygoid muscle, lateral pterygoid muscle, buccinator muscle, and infratemporal region [86,87,88,89]. Excessively deep or incorrectly directed injection may increase the risk of unintended involvement of adjacent masticatory or perioral muscles [68,69,70,87,88,89]. Although the masseter is accessible by palpation, these anatomical relationships indicate that injection into this muscle should not be regarded as technically trivial [86,87,88,89].

A practical safety zone for masseter injection should therefore avoid the extreme anterior border, the posterior–superior region near the parotid gland, and excessively deep placement without clear anatomical rationale [87,88,89] (Figure 1). In selected cases, ultrasonography may help identify muscle thickness, the parotid gland, facial vessels, and anatomical variation [87,88,89], whereas electromyography may help confirm muscle activity, particularly in patients with dystonia or complex movement disorders [54,55,56].

### 3.3. Motor Endplate Zones of the Masseter Muscle

BoNT acts primarily at the neuromuscular junction by inhibiting acetylcholine release from presynaptic motor nerve terminals. Therefore, the distribution of neuromuscular junctions within the target muscle is highly relevant to injection strategy [5,91,92]. Neuromuscular junctions are not uniformly distributed throughout skeletal muscles; rather, they are concentrated within specific regions known as motor endplate zones (MEZs) [57,58].

**Figure 2:**
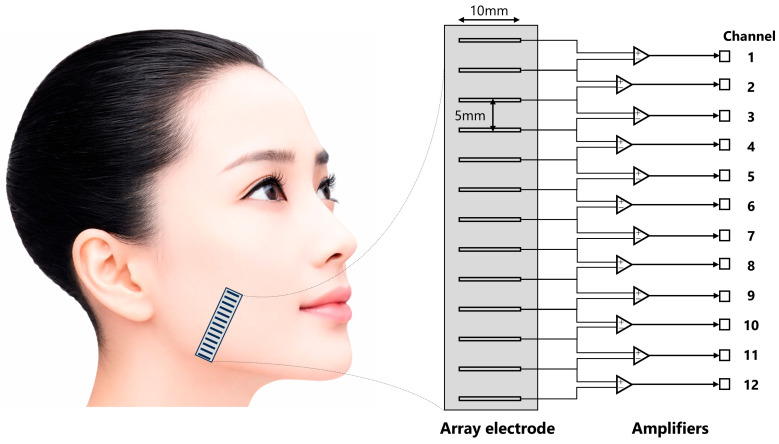
Positional relationship between the array electrode and muscle fibers. Modified from the schematic representation of electrode configuration by Tokunaga [64]. Reproduced with permission from the Japan Prosthodontic Society.

Tokunaga [64] aligned a 13-channel array electrode parallel to the orientation of masseter muscle fibers and fixed it so that the center of the array corresponded to the central portion of the masseter muscle (Figure 2).

**Figure 3:**
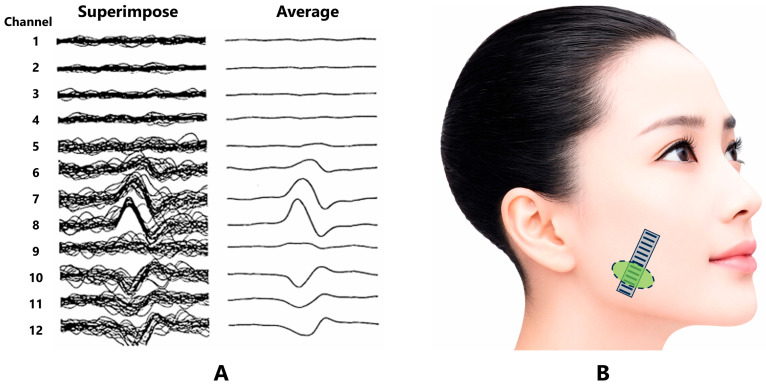
Representative superimposed and averaged EMG spike trains modified from Tokunaga [64] (**A**), and the estimated location of the MEZ (**B**). Reproduced with permission from the Japan Prosthodontic Society.

Using this configuration, the two-dimensional distribution of masseter EMG activity during isometric voluntary contraction was investigated [64]. Identical motor unit action potentials were extracted, and representative examples of their superimposed waveforms and averaged waveforms are shown in Figure 3A.

Analysis of the averaged waveforms revealed a polarity reversal of the EMG signals at channel 9. From this point, the EMG peaks were observed to propagate bilaterally toward both ends of the electrode array with a temporal delay (Figure 3A). In normal muscle, when an endplate potential is generated by a neural impulse, the adjacent muscle fiber membrane is depolarized, resulting in the generation of a muscle action potential [86]. This action potential then propagates from the endplate throughout the muscle fiber. Consistent with this physiological mechanism, symmetric waveforms centered around channel 9 were observed (Figure 3A). Based on these findings, the location of the MEZ in this subject was estimated to be near channel 9 (Figure 3B), positioned slightly inferior to the central portion of the masseter muscle [64].

Electrophysiological and anatomical studies of the human masseter muscle indicate that its MEZs are located within relatively restricted regions of the muscle belly. In surface electrode array studies, motor unit action potentials have been shown to propagate bidirectionally from specific regions, allowing estimation of endplate location [62,63,64]. These findings suggest that the MEZ of the masseter is not distributed diffusely throughout the entire muscle but is concentrated in a band-like region within the central portion of the muscle belly, often corresponding to the lower-central region of the clinically palpable masseter [59,60,61,62,63,64].

The orientation of the MEZ is also relevant. Because MEZs are generally arranged in relation to muscle fiber direction, the distribution of the MEZ may differ between superficial and deep layers of the masseter. The superficial layer, with its oblique fiber orientation, and the deep layer, with more vertical fibers, may not have identical endplate distributions. However, detailed mapping of MEZs in the deep layer of the human masseter remains limited. This uncertainty supports a cautious approach in which MEZ targeting is regarded as an anatomically rational concept rather than a fully validated clinical standard [59,60,61,62,63,64,82,83,84,85].

### 3.4. Implications for BoNT Injection

The anatomical features described above have several implications for BoNT injection into the masseter muscle. First, because the MEZ appears to be located within a relatively restricted region, indiscriminate distribution of BoNT throughout the muscle may not be necessary and may increase the risk of dose-related or diffusion-related adverse effects [54,56,59,60,61,62,63,64]. Second, injections placed too far from the MEZ may require higher doses to achieve a comparable functional effect, potentially increasing unwanted spread [5,54,92]. Third, overly anterior, posterior, or deep injection may affect adjacent muscles or structures rather than the intended functional region of the masseter [80,81,87,88,89].

From a pharmacological perspective, delivery of BoNT close to the MEZ is rational because the neuromuscular junction is the principal site of action [5,91,92]. Nevertheless, direct clinical evidence demonstrating that MEZ-targeted masseter injection is superior to conventional injection techniques remains insufficient [54,59,60,61,62,63,64]. Therefore, the anatomical basis of MEZ targeting should be understood as a rationale for future research and protocol refinement, not as proof of established clinical superiority.

In this review, the anatomical information regarding the masseter muscle, adjacent structures, and MEZ distribution provides the foundation for the later discussion of dose minimization, adverse-event prevention, and a hypothesis-generating MEZ-targeted injection framework.

## 4. Technical Fundamentals of BoNT Injection into the Masseter Muscle

Several BoNT preparations are currently available for clinical use. BoNT type A formulations include onabotulinumtoxinA, abobotulinumtoxinA, incobotulinumtoxinA, and letibotulinumtoxinA, whereas rimabotulinumtoxinB is a botulinum toxin type B formulation [93,94,95]. Because dose units are not interchangeable among different preparations, dose conversion should be interpreted with caution [93,94,95]. In this review, onabotulinumtoxinA is used as the main reference formulation because it has been most extensively used in clinical studies and long-term clinical practice.

The technical goal of masseter BoNT injection is not complete paralysis of the muscle but controlled reduction in excessive or clinically problematic muscle activity while preserving essential masticatory function [3,14,90,96]. Therefore, treatment should generally be initiated with a low dose, and dose adjustment should be individualized according to diagnosis, symptom severity, muscle volume, bite force, baseline muscle activity, previous response to treatment, and risk of adverse effects [3,14,90,96].

For masseter injection, BoNT type A is typically reconstituted with sterile normal saline [93,94,95]. Dilution should be selected to allow accurate intramuscular delivery while avoiding excessive injection volume, which may increase the risk of diffusion into adjacent structures [56,89]. The number of injection points should also be minimized to reduce injection-related pain, bleeding, bruising, and vascular injury [68,70,71,72]. In many clinical settings, three injection points within the functionally relevant portion of the masseter muscle are sufficient, although the number and distribution of sites should be individualized [3,14,52,56,87,88,89].

Needle selection depends on muscle thickness, degree of hypertonicity, patient discomfort, and whether guidance techniques are used [87,88,89]. A 27-gauge needle is generally suitable for masseter injection [3,14]. A thinner needle may reduce insertion pain, but injection resistance may increase, particularly in patients with marked muscle hyperactivity or hypertrophy. Electromyographic needles can be useful when confirmation of muscle activity or deeper layer targeting is required [54,55,56]. Although the masseter muscle is readily accessible by palpation, adjunctive guidance with electromyography or ultrasonography may improve accuracy in selected cases, especially when deep injection, anatomical variation, previous adverse effects, or refractory symptoms are present [54,55,56,87,88,89].

Injection depth should not be standardized without considering individual anatomy [87,88,89]. In clinical practice, superficial injections are often performed at approximately 5–10 mm and deeper injections at approximately 15–20 mm; however, these values should be regarded as practical estimates rather than fixed rules [87,88,89]. Depth should be adjusted according to subcutaneous tissue thickness, masseter volume, sex, age, body habitus, and the intended target layer. Ultrasonography may be particularly useful for assessing muscle thickness and avoiding adjacent structures such as the parotid gland and facial vessels [56,80,81].

As described in the anatomical section, inaccurate placement or excessive diffusion may affect adjacent muscles and structures, including the risorius, zygomaticus major and minor, buccinator, medial pterygoid muscle, parotid gland, facial vessels, and facial nerve branches [80,81,87,88,89]. Therefore, excessively anterior, posterior–superior, or deep injections should be avoided unless there is a clear anatomical and clinical rationale [56,80,81,87,88,89]. Aspiration before injection may be performed to reduce the risk of intravascular injection, and strong massage of the injected area should be avoided because it may promote unintended diffusion [56,80,81].

The duration of the clinical effect of BoNT is usually several months, but the interval between injections should be individualized according to symptom recurrence, functional status, and adverse effects [97,98]. Repeated high-dose injections at short intervals should be avoided to reduce the risk of excessive muscle weakness, atrophy, functional impairment, skeletal unloading, and possible secondary treatment failure due to neutralizing antibodies [97,98]. In patients receiving repeated masseter injections, objective monitoring of bite force, muscle activity, jaw function, and adverse effects is desirable [3,14,75,76,77,78,97,98].

Taken together, these technical considerations emphasize that masseter BoNT injection should be individualized and anatomically informed. These principles provide the practical foundation for the MEZ-targeted framework discussed later, but they should not be interpreted as evidence that any specific injection pattern has already been proven superior in controlled clinical trials.

## 5. Adverse Events and Their Anatomical or Technical Mechanisms

BoNT injection into the masseter muscle is generally considered minimally invasive; however, adverse events may occur when dose, injection site, depth, diffusion, repeated treatment, or patient-specific anatomy and function are not adequately considered [46,50,68,69,70,71,72,73,74,75,76,77,78,79,80,81]. Reported adverse events and their possible anatomical or technical mechanisms are summarized in Table 1. These events should not be regarded as unavoidable consequences of BoNT itself because many are influenced by modifiable technical and clinical factors [46,50,51,68,69,70,71,72,73,74,75,76,77,78,79,80,81].

This mechanistic classification is intended to support interpretation of previous studies and the design of safer, more standardized future protocols.

### 5.1. Dose-Related Adverse Events

Table 2 summarizes the variability of target muscles, BoNT formulations, dose, and total bilateral dose across randomized trials. Protocols ranged from masseter-only injection to multi-muscle approaches, and total bilateral doses ranged from approximately 40 U to 200 U. Because dose units are product-specific, the formulation used in each study is clinically relevant and should be reported alongside the dose.

Dose-related adverse effects are particularly relevant during repeated treatment. Excessive or repeated high-dose injections may cause masticatory weakness, reduced bite force, reduced occlusal force, decreased masticatory performance, masseter atrophy, and potentially reduced mandibular loading [50,51,75,76,77,78,79,90,96].

For this reason, dose minimization is a central safety principle. The goal is not complete paralysis but controlled reduction in clinically problematic activity while preserving essential masticatory function. Baseline bite force, muscle volume, symptom severity, and functional impairment should guide dose selection and follow-up [3,14,90,96].

### 5.2. Diffusion-Related Adverse Events

Diffusion-related events include facial asymmetry, asymmetric smile, xerostomia, dysphagia, and speech changes [15,16,68,69,70,87,88,89]. These events are influenced by injection site, depth, direction, dilution, volume, and whether adjacent masticatory, perioral, lingual, or salivary structures are exposed to toxin spread.

### 5.3. Injection-Related Adverse Events

Injection-related events include injection-site pain, bruising, swelling, hematoma, and transient sensory discomfort [68,70,71,72]. These are usually mild and self-limited but may be influenced by needle size, number of insertion points, vascular anatomy, anticoagulant use, and technique.

### 5.4. Paradoxical Masseter Bulging

Paradoxical masseter bulging is a distinctive adverse effect in which localized prominence appears after BoNT injection, often during clenching. It is thought to result from uneven weakening of different masseter layers, compartments, or fascicles [80,81]. Understanding the layered architecture of the masseter is therefore important when selecting the depth and distribution of injection.

### 5.5. Skeletal and Long-Term Functional Considerations

Mandibular bone changes have been reported after repeated or high-dose masseter BoNT injection, probably related at least in part to reduced masticatory force and skeletal loading [50,51,75,76,77,78,79]. Their clinical significance, reversibility, and dose–response relationship remain incompletely understood; therefore, long-term repeated treatment should be accompanied by reassessment of indication, dose, treatment interval, bite force, masticatory function, and patient-reported benefit.

### 5.6. Implications for Anatomically Informed Injection

Overall, adverse-event prevention depends on accurate diagnosis, appropriate indication, dose minimization, individualized depth, avoidance of high-risk anatomical regions, careful functional monitoring, and transparent reporting of injection protocols [3,14,46,50,51,54,68,69,70,71,72,73,74,75,76,77,78,79,80,81,87,88,89,90,96].

MEZ-targeted injection is relevant to this safety framework because BoNT acts at the neuromuscular junction, and masseter neuromuscular junctions are concentrated within relatively restricted regions [5,57,58,59,60,61,62,63,64,91,92]. Delivering BoNT closer to these regions may theoretically reduce unnecessary dose and diffusion, but clinical superiority and improved safety remain unproven [54].

## 6. Current Evidence and Methodological Limitations

Clinical evidence regarding BoNT injection into the masseter muscle differs substantially according to the clinical condition being treated. In oromandibular dystonia, BoNT is widely used as a therapeutic option for reducing involuntary jaw-closing muscle activity and improving oral function [3,14,15,16]. In contrast, evidence for BoNT in masseter-related myalgia, myogenous TMD, and bruxism-associated masticatory muscle pain remains more heterogeneous and controversial [33,34,35,36,37,38,39,40,41,42,43,44,45,46,47,48,49,50,51,52,53]. This difference reflects not only variation in disease mechanisms but also differences in diagnostic criteria, patient selection, outcome measures, injection protocols, and treatment goals [3,10,11,12,13,14,52,66].

In patients with jaw-closing dystonia, the therapeutic target is excessive involuntary muscle contraction [3,14,15,16]. Therefore, a reduction in masseter activity is directly related to the pathophysiology of the disorder. However, even in this context, treatment outcomes depend on accurate identification of the involved muscles, appropriate dose selection, injection depth, and avoidance of unnecessary diffusion [3,15,16]. Most clinical reports on oromandibular dystonia are based on observational studies or accumulated clinical experience, and controlled trials specifically comparing different masseter injection strategies, including MEZ-targeted approaches, remain limited [3,14,15,16].

For myogenous TMD and masseter-related myalgia, the evidence is less consistent [33,34,35,36,37,38,39,40,41,42,43,44,45,46,47,48,49,50,51,52,53]. Several randomized controlled trials and clinical studies have reported pain reduction after BoNT injection [4,20,24,25,29], whereas others have found no clear superiority over placebo or conservative treatment [17,19,21,22,23]. These inconsistent findings should be interpreted in light of the heterogeneous nature of TMDs. Because TMD is not a single diagnosis, studies that include different combinations of myalgia, arthralgia, disc displacement, headache, psychosocial factors, central sensitization, or other orofacial pain conditions may not evaluate the same clinical entity [10,65]. If the masseter muscle is not the principal source of pain, masseter-directed chemodenervation is unlikely to produce consistent benefit [10,52].

Similarly, interpretation of BoNT studies in bruxism requires caution. Bruxism is a masticatory muscle activity or behavior rather than a disease [11,12,13]. Therefore, the presence of bruxism alone does not necessarily indicate a need for treatment [11,12,13]. Some studies have evaluated the reduction in electromyographic activity or jaw motor events, whereas others have focused on pain, tooth wear, muscle fatigue, or patient-reported symptoms [66]. These outcomes reflect different clinical questions. BoNT may reduce the intensity of masseter contraction, but this should not be equated with treatment of bruxism itself. The more appropriate target is clinically significant bruxism-associated masticatory muscle pain or functional impairment in selected patients [11,12,13,14,66].

Previous studies used highly variable injection protocols, including masseter-only injection, combined masseter and temporalis injection, and multi-muscle approaches involving the pterygoid muscles (Table 2). Dose, formulation, dilution, volume, number of injection points, depth, target layer, and retreatment interval also varied or were incompletely reported. Therefore, interventions labeled as “BoNT injection into the masseter” may represent substantially different procedures [14,19,20,21,22,23,24,25,26,27,28,29,30,31,32,52].

Another limitation is insufficient reporting of injector training, anatomical localization, and the use of guidance techniques. These factors may influence both efficacy and adverse events but are inconsistently described in masticatory muscle studies [52,54,55,56,87,88,89].

Most previous trials also did not consider MEZ distribution within the masseter muscle. Because BoNT acts at the neuromuscular junction, endplate distribution may contribute to variability in response, although this remains a hypothesis requiring prospective validation [5,54,59,60,61,62,63,64,91,92].

Adverse-event interpretation is limited by inconsistent reporting of dose, depth, diffusion, concomitant muscle injection, repeated treatment, and patient-specific anatomy. Future studies should use standardized adverse-event terminology and report severity, duration, functional impact, and presumed mechanism [46,50,51,68,69,70,71,72,73,74,75,76,77,78,79,80,81].

Systematic reviews and umbrella reviews are useful, but their conclusions are constrained by heterogeneity in primary studies [33,34,35,36,37,38,39,40,41,42,43,44,45,46,47,48,49,50,51]. Broadly pooled results should not be overgeneralized either to dismiss carefully selected anatomically guided injections or to support indiscriminate use in all TMD or bruxism populations.

Future studies should clearly define clinical populations, report BoNT preparation and full injection parameters, use condition-specific functional outcomes, and include long-term adverse-event monitoring [3,10,11,12,13,14,15,52,54,55,56,66,87,88,89,93,94,95,96].

Taken together, the current evidence does not support the indiscriminate use of BoNT for TMDs or bruxism [10,11,12,13,65,66,67]. At the same time, it does not exclude a potential role for BoNT in carefully selected conditions involving excessive or painful masseter activity. The main limitation of the existing literature is that BoNT injection has often been evaluated as a uniform intervention despite substantial heterogeneity in diagnosis and technique [52,54,55,56]. This provides the rationale for developing and testing a more anatomically informed, dose-minimized, MEZ-targeted framework in future controlled studies.

## 7. Rationale for MEZ-Targeted Low-Dose Injection

The rationale for MEZ-targeted BoNT injection is based on the pharmacological site of action of BoNT and the non-uniform distribution of neuromuscular junctions. Because BoNT inhibits acetylcholine release at presynaptic motor terminals, injection site selection may influence the efficiency of neuromuscular blockade [5,57,58,91,92].

In the masseter muscle, anatomical and electrophysiological studies suggest that MEZs are located within relatively restricted regions of the muscle belly [59,60,61,62,63,64]. Targeting the estimated MEZ may therefore allow more efficient access to neuromuscular junctions and potentially reduce the dose required for a functional effect, although this remains unproven clinically [5,54,92].

A low-dose strategy is important because the masseter is essential for mastication, jaw stabilization, and occlusal function. Excessive weakening may reduce bite force, impair chewing, promote atrophy, alter facial contour, and decrease mandibular loading [75,76,77,78,79,90,96].

MEZ-targeted injection may be relevant for jaw-closing dystonia and selected cases of masseter-related myalgia or bruxism-associated masticatory muscle pain; however, comparative studies are needed to test whether it improves dose efficiency, efficacy, or safety [3,10,11,12,13,14,15,52,54,59,60,61,62,63,64].

The layered structure of the masseter must also be considered. MEZ targeting is not simply injection into a fixed surface point but integration of surface landmarks, clenching palpation, muscle thickness, target layer, depth, and guidance when appropriate [54,55,56,82,83,84,85,86,87,88,89].

In this context, the MEZ-targeted concept represents a research hypothesis: using the minimum effective dose at a relevant anatomical target while limiting unnecessary exposure of non-target areas [54,59,60,61,62,63,64,90,96].

## 8. Proposed Research Framework for Future Studies

This section outlines a proposed research framework for future studies of MEZ-targeted masseter BoNT injection. It is intended to standardize study design and reporting, not to provide a routine clinical protocol.

The clinical aim should be defined by condition: reducing involuntary excessive contraction in jaw-closing dystonia or modulating clinically relevant painful or excessive masseter activity in selected myalgia or bruxism-associated pain populations [3,10,11,12,13,14,15,52].

### 8.1. Patient Selection and Pretreatment Assessment

Future studies should stratify participants by condition because jaw-closing dystonia, masseter-related myalgia, and bruxism-associated masticatory muscle pain differ in pathophysiology, treatment goals, and expected outcomes [3,10,11,12,13,14,15,66,96]. Pretreatment assessment should document symptomatic muscles, pain location, jaw function, bite force when available, baseline masticatory performance, and previous conservative care [10,14,52,65,96].

### 8.2. Identification of the Target Region

The target region should be selected using anatomical landmarks and functional assessment, such as palpation during clenching to identify the masseter belly and its thickest portion [54,55,56,87,88,89]. The estimated MEZ often corresponds to the lower-central portion of the palpable masseter, but its precise location may vary among individuals and layers [59,60,61,62,63,64,87,88,89] (Figure 3).

### 8.3. Injection Points and Needle Direction

**Figure 4:**
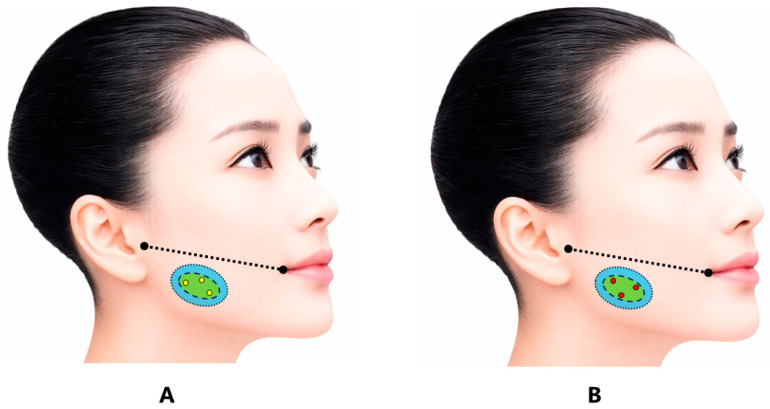
Schematic MEZ-targeted framework for masseter BoNT injection. The estimated MEZ is shown in green, the safety area in light blue, and potential injection points in yellow (**A**) or red (**B**). The figure is schematic and should not be interpreted as a validated clinical protocol. The black dotted line connects the oral commissure and the tragus and indicates the upper boundary of the generally recommended injection region; injections are typically placed below this line to reduce the risk of diffusion into adjacent facial muscles.

Future protocols may test a limited number of injection points within the estimated MEZ region, such as a three-point framework in the lower-central masseter belly (Figure 4), while recognizing that this pattern has not been validated [54,59,60,61,62,63,64,68,70,71,72]. Studies should report skin site, needle direction, and whether insertion points are repeated or alternated between sessions.

### 8.4. Injection Depth and Target Layer

Injection depth should be individualized according to subcutaneous tissue, masseter thickness, target layer, and clinical objective [87,88,89]. Studies should distinguish numerical needle depth from the intended anatomical layer, because the same depth may reach different layers in different patients.

Because uneven layer involvement may contribute to paradoxical bulging or inadequate response, future studies should report skin site, depth, target layer, needle direction, and guidance method [54,55,56,80,81,82,83,84,85,86,87,88,89].

### 8.5. Dose and Dilution

Future studies should test dose-minimized strategies while preserving masticatory function. Dose should be reported by product, per side, total dose, dilution, volume, muscle size, baseline bite force, and clinical indication [3,14,75,76,77,78,79,90,93,94,95,96].

### 8.6. Guidance Techniques

Guidance methods should be reported consistently. Electromyography may confirm muscle activity, whereas ultrasonography may document muscle thickness, layer structure, adjacent structures, and needle depth [54,55,56,87,88,89].

### 8.7. Safety Monitoring and Outcome Assessment

Outcome assessment should include condition-specific endpoints, objective functional measures, patient-reported outcomes, duration of effect, and standardized adverse-event reporting [3,10,14,46,50,51,66,68,69,70,71,72,73,74,75,76,77,78,79,80,81,99,100].

### 8.8. Proposed Framework

The proposed research framework can be summarized as follows: stratify patients by diagnosis; define the clinical target; estimate the MEZ region using palpation, landmarks, and guidance when needed; use a limited number of points within an anatomical safety zone; individualize depth and layer; use the minimum effective dose; and monitor function and adverse events (Table 3).

## 9. Conclusions

BoNT injection into the masseter muscle should be understood as a muscle-directed intervention for selected conditions involving excessive or painful masseter activity, rather than as a generic treatment for TMDs or bruxism. These conditions differ in pathophysiology, treatment goals, and outcome measures and should be evaluated separately [3,10,11,12,13,14,15,66].

MEZ-targeted masseter injection is anatomically and pharmacologically rational because BoNT acts at neuromuscular junctions concentrated within motor endplate zones [5,57,58,59,60,61,62,63,64,91,92]. Targeting the estimated MEZ with a dose-minimized approach may theoretically improve dose efficiency and reduce unnecessary exposure, but direct evidence of superiority remains insufficient [54,57,58,59,60,61,62,63,64,90,96].

Accordingly, MEZ-targeted masseter BoNT injection should be regarded as a hypothesis-generating research framework. Future studies should use clearly defined populations, standardized diagnoses, complete reporting of BoNT formulation and injection technique, objective functional outcomes, and systematic adverse-event assessment [3,10,11,12,13,14,15,52,54,55,56,66,87,88,89,90,93,94,95,96].

## Figures and Tables

**Table 1:** Adverse events associated with masseter BoNT injection, possible anatomical or technical mechanisms, and preventive considerations.

Adverse Events	Possible Anatomical or Technical Mechanisms	Preventive Considerations
Masticatory weakness, reduced bite force, reduced occlusal force, and reduced masticatory performance [10,11,64,65]	Excessive dose, excessive reduction in jaw-closing muscle activity, repeated high-dose injections, or diffusion into adjacent masticatory muscles	Use the minimum effective dose. Assess baseline bite force, muscle volume, EMG activity, and masticatory function when possible. Dose reduction should be considered in patients with low bite force, elderly patients, women, and patients without masseter hypertrophy.
Masseter muscle atrophy [11,65,74,75,76,77]	Dose-dependent muscle weakening, repeated injections, reduced functional loading, and long-term suppression of masseter activity	Avoid unnecessary high-dose or short-interval repeated injections. Reassess indication, dose, treatment interval, muscle volume, and functional benefit before each retreatment.
Mandibular bone changes, including decreased cortical thickness, bone density, or bone volume [44,45,74,75,76,77]	Reduced masticatory force and skeletal unloading after repeated or high-dose masseter weakening	Avoid repeated high-dose injections, especially in patients with low baseline bite force or without masseter hypertrophy. Monitor bite force, masticatory function, and long-term skeletal or facial contour changes when treatment is repeated.
Facial asymmetry or asymmetric smile [13,15,20,66,72,73]	Diffusion into facial expression muscles, including the risorius, zygomaticus major and minor, depressor anguli oris, or lateral orbicularis oris	Avoid excessively anterior injection sites. Keep injection points within the masseter belly and use low injection volume. Consider ultrasound guidance when anatomy is uncertain.
Dysphagia [66,67,68,69]	Deep diffusion or excessive dose affecting adjacent deep muscles, the medial pterygoid muscle, suprahyoid muscles, or floor-of-mouth muscles; increased risk with multi-muscle injections	Avoid excessively deep injection without a clear rationale. Use cautious dosing, especially when injecting multiple masticatory, lingual, or suprahyoid muscles. Stage treatment when necessary.
Speech changes [66,67,68,69]	Diffusion to adjacent perioral, lingual, suprahyoid, or deep masticatory muscles; excessive weakening in multi-muscle treatment	Individualize dose and depth. Avoid unnecessary diffusion to non-target muscles. Carefully evaluate speech and swallowing function in patients with oromandibular dystonia or complex movement disorders.
Xerostomia [10,55,66,68]	Diffusion toward the parotid gland or structures related to parasympathetic innervation, including the auriculotemporal nerve region	Avoid posterior–superior injection sites and excessive posterior placement. Consider placing posterior injections sufficiently anterior to the posterior border of the masseter. Ultrasound may be useful to identify parotid extension.
Perioral swelling, bruising, or hematoma [66,68,70]	Vascular injury, repeated needle passes, local inflammation, or hematoma formation; facial artery and vein are located near the anterior border of the masseter	Minimize the number of insertion points and needle passes. Avoid excessively anterior injection. Use gentle technique and consider vascular anatomy, anticoagulant use, and ultrasound guidance in selected cases.
Paresthesia, numbness, or sensory discomfort [66,70,71]	Local edema, hematoma, inflammatory response, transient compression of sensory branches, or modulation of sensory neuropeptide release	Minimize injection volume and unnecessary needle trauma. Avoid vascular injury and excessive local tissue inflammation. Explain the possibility of transient sensory symptoms before treatment.
Paradoxical masseter bulging [79,80]	Uneven weakening of different layers, compartments, or fascicles of the masseter; excessively deep or uneven injection; anatomical compartmentalization	Understand the layered structure of the masseter. Avoid excessively deep or uneven injection. If bulging occurs, consider observation, dose adjustment, modified injection depth, or carefully targeted additional injection. EMG or ultrasound may be helpful in selected cases.
Eye drooping or periocular imbalance [48]	Possible diffusion or functional imbalance when masseter and temporalis injections are performed simultaneously; unintended involvement of muscles around the orbit is rare but possible	Avoid excessive dosing and unintended diffusion during combined masseter–temporalis treatment. Confirm target muscle and injection depth carefully.
Injection-site pain or discomfort [66,68,70]	Needle insertion pain, local tissue trauma, injection pressure, or inflammatory response	Use an appropriate needle gauge, gentle technique, and the minimum necessary number of injection points. Avoid excessive injection pressure and unnecessary repeated puncture.

**Table 2:** Variability in BoNT formulations and injection protocols across randomized controlled trials involving BoNT injection into the masticatory muscles.

Study and BoNT Formulation	Muscles Injected (*n*)	Dose (Per Side)	Total Dose (Bilateral)
Nixdorf et al. [19] BoNT-A (specific formulation not reported)	M, T (10)	M: 50 U, T: 25 U	150 U
Guarda-Nardini et al. [20] onabotulinumtoxinA (Botox)	M, T (10)	M: 30 U, T: 20 U	100 U
Kurtoglu et al. [21] BoNT-A (specific formulation not reported)	M, T (12)	M: 30 U, T: 20 U	100 U
Ernberg et al. [17] BoNT-A (specific formulation not reported)	M (12)	M: 50 U	100 U
Shim et al. [2] BoNT-A (specific formulation not reported) BoNT-A (specific formulation not reported)	M (10)	M: 25 U	50 U
	M, T (10)	M: 25 U, T: 25 U	100 U
De Carli et al. [22] BoNT-A (specific formulation not reported)	M, T (10)	Not clearly defined	Variable
Patel et al. [23] incobotulinumtoxinA	M, T, Lpt (15)	M: 50 U, T: 25 U, Lpt: 10 U	170 U
Al-Wayli et al. [24] BoNT-A (specific formulation not reported)	M (11)	M: 20 U	40 U
Jadhao et al. [25] BoNT-A (specific formulation not reported)	M, T (10)	M: 60 U, T: 40 U	200 U
Ondo et al. [26] onabotulinumtoxinA	M, T (13)	M: 30 U, T: 20 U	100 U
Shim et al. [27] BoNT-A (specific formulation not reported)	M (10)	M: 25 U	50 U
De la Torre Canales et al. [4] onabotulinumtoxinA (Botox) onabotulinumtoxinA (Botox) onabotulinumtoxinA (Botox)	M, T (20)	M: 30 U, T: 10 U	80 U
	M, T (20)	M: 50 U, T: 20 U	140 U
	M, T (20)	M: 75 U, T: 25 U	200 U
Montes-Carmona et al. [28] BoNT-A (specific formulation not reported)	M, T, Lpt, Mpt (12)	M: 24–30 U, T: 24 U, Lpt: 8 U, Mpt: 8 U	100–150 U

M, masseter; T, temporalis; Lpt, lateral pterygoid; Mpt, medial pterygoid; U, units. BoNT formulation is listed as reported in each study; when the specific commercial preparation was not clearly stated in the accessible report, it is indicated as not specified. Dose units are product-specific and should not be directly converted across BoNT preparations.

**Table 3:** Hypothesis-generating framework for MEZ-targeted BoNT injection into the masseter muscle.

Parameter	Proposed Framework	Rationale
Clinical target	Selected patients with clinically relevant excessive or painful masseter activity, including jaw-closing oromandibular dystonia, masseter-related myalgia, and bruxism-associated masticatory muscle pain	Avoids presenting BoNT as a generic treatment for TMDs or bruxism. TMDs are heterogeneous conditions, and bruxism is a masticatory muscle activity or behavior rather than a disease entity [10,11,12,13].
Treatment objective	Controlled reduction in clinically problematic masseter activity while preserving essential masticatory function	Complete paralysis is unnecessary and may cause masticatory weakness, reduced bite force, muscle atrophy, and functional impairment [3,14,15,50,51,75,76,77,78].
Target region	Estimated MEZ in the central or lower-central region of the masseter muscle belly	BoNT acts at the neuromuscular junction, and neuromuscular junctions are concentrated within motor endplate zones rather than being uniformly distributed throughout the muscle [5,57,58,91,92].
MEZ localization	Estimate the target region using palpation during clenching, surface anatomical landmarks, and available anatomical or electrophysiological information	Surface EMG and anatomical studies suggest that the human masseter MEZ is concentrated in a restricted band-like region within the muscle belly [59,60,61,62,63,64].
Injection points	Use a limited number of divided injections within the estimated MEZ region; a three-point framework may be practical in many cases	Divided injections may help distribute BoNT within the functionally relevant muscle region while avoiding unnecessary injection sites and excessive tissue trauma. This remains a hypothesis-generating approach rather than a validated standard [54,56,59,60,61,62,63,64].
Areas to avoid	Avoid the extreme anterior border, posterior–superior region, and excessively deep placement unless clearly justified	Anterior diffusion may affect facial expression muscles and cause smile asymmetry; posterior–superior diffusion may involve the parotid gland; excessive depth may affect adjacent deep structures [56,80,81,87,88,89].
Needle direction	Central point may be inserted perpendicular to the skin; anterior and posterior points may be directed slightly toward the central muscle belly	Inward needle orientation is intended to keep BoNT within the masseter belly and reduce unintended spread outside the target muscle. This technical concept requires future validation [56,87,88,89].
Injection depth	Individualize depth according to muscle thickness, subcutaneous tissue, sex, body habitus, hypertrophy, and intended target layer	The masseter has layered anatomy and variable thickness. Fixed-depth injection may be inappropriate across patients; ultrasonography may help assess muscle thickness and adjacent structures [82,83,84,85,86,87,88,89].
Target layer	Superficial or central injection may be sufficient in many patients; deeper injection may be considered in selected cases with marked hypertrophy, strong jaw-closing dystonia, or inadequate response	Superficial and deep portions may differ in fiber direction, function, and diffusion risk. Detailed mapping of deep-layer MEZs remains limited, so deeper injection should be cautious [82,83,84,85,86,87,88,89].
Dose strategy	Begin with the minimum effective dose and escalate cautiously only when clinically necessary	Dose minimization is important to preserve mastication and reduce dose-related adverse effects, including weakness, atrophy, reduced bite force, and possible skeletal unloading [3,14,15,50,51,75,76,77,78,90].
BoNT preparation	Clearly report the BoNT product used; dose units should not be assumed to be interchangeable across formulations	BoNT preparations differ pharmacologically, and dose conversion between products should be interpreted cautiously [93,94,95].
Dilution and injection volume	Report dilution and volume per injection point; avoid unnecessarily large injection volumes	Dilution and injection volume may influence diffusion and spread. Excessive diffusion is relevant to adverse events and should be controlled and reported [54,56,69,70].
Guidance techniques	EMG or ultrasonography may be considered in selected cases, especially for dystonia, deep injection, anatomical variation, previous adverse effects, or refractory symptoms	EMG may confirm muscle activity; ultrasonography may identify muscle thickness, the parotid gland, facial vessels, and needle depth [55,87,88,89].
Outcome assessment	Evaluate pain, dystonia severity, jaw function, bite force, masticatory performance, EMG activity, quality of life, patient satisfaction, and duration of effect	Outcomes should match the clinical condition and should not be limited to pain intensity alone, especially when oromandibular dystonia, myalgia, and bruxism-associated symptoms are studied separately [3,10,11,12,13,14,15,66].
Adverse-event monitoring	Systematically assess masticatory weakness, reduced bite force, facial asymmetry, xerostomia, dysphagia, speech changes, paradoxical bulging, muscle atrophy, and mandibular bone changes	Reported adverse events may be dose-related, diffusion-related, injection-related, or related to repeated high-dose treatment [37,46,50,51,68,69,70,71,72,73,74,75,76,77,78,79,80,81].
Long-term follow-up	Monitor cumulative dose, retreatment interval, bite force, muscle volume, facial contour, mandibular bone changes, and functional benefit during repeated treatment	Repeated or high-dose injections may affect masseter volume, masticatory loading, and mandibular bone structure; neutralizing antibodies are also a consideration in repeated BoNT therapy [50,51,75,76,77,78,79,97,98].
Research status	Treat the MEZ-targeted framework as a testable research model, not as an established clinical guideline	Direct clinical evidence demonstrating the superiority of MEZ-targeted masseter injection over conventional landmark-based injection remains insufficient and should be tested in future controlled studies [54,57,58,59,60,61,62,63,64].

This framework is intended for future clinical studies and should not be interpreted as an established guideline. MEZ, motor endplate zone; BoNT, botulinum neurotoxin; EMG, electromyography.

## Data Availability

The original contributions presented in this study are included in the article. Further inquiries can be directed to the corresponding author.

## Abbreviations

The following abbreviations are used in this manuscript:

BoNT	Botulinum neurotoxin
TMD	Temporomandibular disorder
MEZ	Motor endplate zone
